# Effect of Bazi Bushen Capsule on D‐Galactose‐Induced Human Endothelial Cell Senescence Through PI3K/Akt/eNOS Signaling Pathway

**DOI:** 10.1002/agm2.70031

**Published:** 2025-06-11

**Authors:** Lulu Yao, Fuyao Li, Jingnian Ni, Mingqing Wei, Ting Li, Jing Shi, Jinzhou Tian

**Affiliations:** ^1^ Department of Neurology Dongzhimen Hospital of Beijing University of Chinese Medicine Beijing China; ^2^ Beijing University of Chinese Medicine Beijing China

**Keywords:** aging, Bazi Bushen capsule, cell senescence, PI3K/Akt/eNOS, traditional Chinese medicine

## Abstract

**Objectives:**

BaZi BuShen capsule (BZBS) is a Chinese herbal prescription with the function of nourishing the kidney, replenishing essence, and combating aging. This study aims to investigate the effects of BZBS on aging endothelial cells and to elucidate the underlying mechanisms.

**Methods:**

An aging human brain microvascular endothelial cells (HBMECs) model was established using D‐galactose (D‐gal) for 24 h. The efficacy of the model was evaluated by the positive rate of senescence‐associated β‐galactosidase (SA‐β‐Gal) staining. The treatment was administered using drug‐containing serum of BZBS. The experimental groups comprised the following: Control, Model, and groups treated with drug‐containing serum at low (BZBSL), medium (BZBSM), and high (BZBSH) doses of BZBS, in addition to a pathway inhibitor group (LY294002 [5 μM/L]). Western blotting and immunofluorescence assays were conducted to evaluate the expression levels of proteins. Nitric oxide (NO) levels in the cells were detected using an Assay Kit. The experiments were independently repeated five times.

**Results:**

D‐gal significantly elevated the SA‐β‐Gal positive rate in HBMECs. Intervention with BZBS significantly reduced the percentage of SA‐β‐Gal positive cells (*p* < 0.001). Compared to the Model group, drug‐containing serum of BZBS significantly increased the expression levels of PI3K, p‐Akt, Akt, and eNOS (*p* < 0.010) and elevated NO levels in the cells (*p* < 0.010), by which BZBS ameliorated HBMECs aging and enhanced the function of aging HBMECs.

**Conclusions:**

Our findings indicate that BZBS can mitigate D‐gal‐induced aging in HBMECs by activating the PI3K/Akt/eNOS signaling pathway.

## Graphical abstract

Bazi Bushen capsule (BZBS) is an over‐the‐counter drug approved by China FDA with the function of nourishing the kidney, replenishing essence, and combating aging. In this study, we concluded that the PI3K/AKT/eNOS signaling pathway contributed to BZBS repair of endothelial dysfunctions induced by D‐galactose.

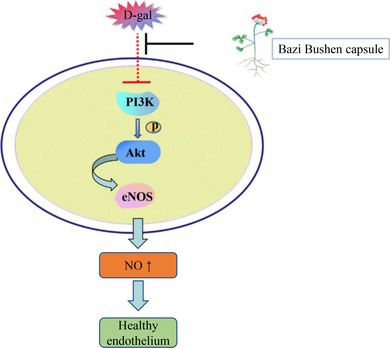

## 1 Introduction

Senescence refers to a decrease in function at the cellular and organismal levels due to the occurrence of various physiological or pathological changes [1]. Senescence is the cause of many age‐related diseases, which can lead to a gradual decline in body function and increase the risk of age‐related diseases [2]. In the elderly, the augmented production of senescent cells coupled with diminished clearance mechanisms results in their accumulation, thereby impairing physiological functions [3, 4].

One of the key components of the body that is affected by aging is the endothelial cells. As critical components of the blood–brain barrier, brain endothelial cells undergo functional decline with aging, which can cause endothelial dysfunction and subsequent vascular impairment. Consequently, maintaining the function of the endothelial cells could be an effective way to delay aging [5]. Vascular endothelial cells secrete a variety of vasoactive substances, including the vasodilator nitric oxide (NO), whose diminished bioavailability is a hallmark of impaired vasodilation. Endothelial nitric oxide synthase (eNOS) within endothelial cells facilitates the production of NO, with its activity and expression modulated by the PI3K/Akt pathway [6]. Inhibition of the PI3K/Akt/eNOS signaling pathway can attenuate eNOS activity and NO synthesis, thereby contributing to endothelial cell dysfunction.

Notably, Chinese herbal medicines and extracts can act on diverse molecular targets in an additive manner or even synergistically in mitigating and treating age‐related disease [7]. Several small molecules originating from herbal medicines have been proven to be effective in delaying aging [8, 9, 10, 11]. Quercetin was found to selectively clear senescent cells and to improve cognitive function [12, 13]. Quercetin is designated as a senolytic due to its ability to prolong healthy lifespan and reduce the likelihood of developing age‐related diseases by selectively killing senescent cells [14]. More recently, fisetin has been identified as a new senolytic that is capable of protecting neurons via anti‐inflammatory processes and elimination of cellular senescence [15, 16]. Accordingly, Traditional Chinese Medicine (TCM) presents a potential alternative due to its properties in preventing aging. Based on the theory of TCM, kidney deficiency is recognized as a fundamental pathogenic mechanism underlying the aging process. BaZi BuShen capsule (BZBS), derived from the Tang Dynasty famous Wuzi Yanzong pill, is an over‐the‐counter drug approved by China FDA (No. B20020585) with the function of nourishing the kidney, replenishing essence, and combating aging. BZBS consists of 16 botanical drugs; the specific ingredients are shown in Table S1. As described previously, 14 compounds have been identified in BZBS by ultra‐performance liquid chromatography (UPLC) analysis, 11 of which were phytoestrogens (PEs) [17]. These identified compounds may underlie the beneficial effects of BZBS to protect against aging. Previous studies indicated that BZBS can improve the degeneration of testicular morphology and spermatogenesis by regulating the Sirt6/NF‐jB and Sirt6/P53 pathways in aging mice [18]. It also can ameliorate brain function decline in aging mice by protecting redox homeostasis, telomere integrity, apoptosis inhibition, and activation of the Sirt6/P53‐PGC‐1a‐TERT and Sirt6/NRF2/HO‐1 signaling pathway [19]. Additionally, BZBS is effective at protecting mesenchymal stem cells against D‐gal‐induced senescence by regulating cell cycle regulation via the Cyclin D1/CDK4/E2F1 signaling pathway [20].

However, there are few reports on the potential pharmacological functions of BZBS in improving aging endothelial cells. Therefore, the present study aimed to verify whether BZBS has a protective effect on D‐gal‐induced senescent endothelial cells by the PI3K/Akt/eNOS signaling pathway.

## 2 Materials and Methods

### 2.1 Reagents and Instruments

BZBS powder (A1605001) was provided by Shijiazhuang Yiling Pharmaceutical Co. Ltd. As described previously, the preparation of BZBS has been described in sufficient detail [18, 21]. D‐gal (Cat# G0750) was purchased from Sigma‐Aldrich (St. Louis, MO, USA). DMEM High Glucose Medium (AG29719232) was obtained from Hyclone (Logan, Utah, USA). Endothelial Cell Medium (1001) was acquired from ScienCell (San Diego, California, USA). Trypsin solution and fetal bovine serum (25200072, 12483020) were obtained from Gibco (New York, USA). The Senescence‐Associated β‐Galactosidase (SA‐β‐Gal) Assay Kit (C0602), BCA Protein Assay Kit (P0010), Total Nitric Oxide Assay Kit (S0024), and Immunostaining Permeabilization Solution (P0095) were purchased from Beyotime (Shanghai, China). The CCK‐8 Assay Kit (AQ‐308) was obtained from Analysis Quiz (New York, USA). The SuperSignal ECL Chemiluminescent Substrate Kit (PK10003) and Protein Pre‐stained Marker (PL00002) were purchased from Proteintech bio‐company (Wuhan, China). The Omni‐Easy One‐Step PAGE Gel Preparation Kit (PG212) was obtained from Yamei. LY294002 (HY‐10108) was purchased from MedChemExpress (New Jersey, USA). The primary antibody was acquired from Abcam (Cambridge, UK). Alexa Fluor 488 and Alexa Fluor 594 were obtained from Bioss (Beijing, China). Information on all reagents and antibodies is shown in Table S2 for the purpose of replicating procedures and results.

### 2.2 Cell Culture

Human brain microvascular endothelial cells (HBMECs) were procured from Shanghai Zhongqiao Xinzhou Biotechnology Co. Ltd. The HBMECs were maintained in endothelial cell medium supplemented with 5% fetal bovine serum, 1% penicillin/streptomycin, and 1% endothelial cell growth factor (ECGF) at 37°C and 5% CO_2_. The culture medium was refreshed daily, and subculturing was performed upon reaching 80% confluence.

### 2.3 Preparation of Drug‐Containing Serum of BZBS

Eighty male Sprague–Dawley (SD) rats of specific pathogen‐free (SPF) grade were procured from Beijing Vital River Laboratory Animal Technology Co. Ltd. (License No. SYXK (Jing) 2023‐0011). Eighty SD rats were randomly allocated into four experimental groups: a saline control group, a low‐dose BZBS group, a medium‐dose BZBS group, and a high‐dose BZBS group. The rats were administered oral gavage doses equivalent to 6.3 times the adult human equivalent dose according to the human–rat dose translation on a body‐surface‐area basis. The low‐dose group received a clinical dose equivalent of 0.126 g/kg/day, while the medium and high‐dose groups received doses at 2‐ and 4‐fold compared with the low‐dose group. The gavage was administered twice daily over a duration of 7 days. Whole blood was taken from the abdominal aorta of rats, and the serum was separated by centrifugation at 3000 rpm, 4°C for 15 min. The serum was then filtered using a 0.22 μm membrane (Millipore) and stored at −80°C.

All experiments were approved by the Animal Ethics Review Board of Beijing University of Chinese Medicine (Ethics Review No. 2023‐041307‐2102). Efforts were made to minimize the number of animals utilized and limit discomfort, pain, or any other suffering of the experimental animals in this study.

### 2.4 Establishment of the Senescence Cell Model

**FIGURE 1:**
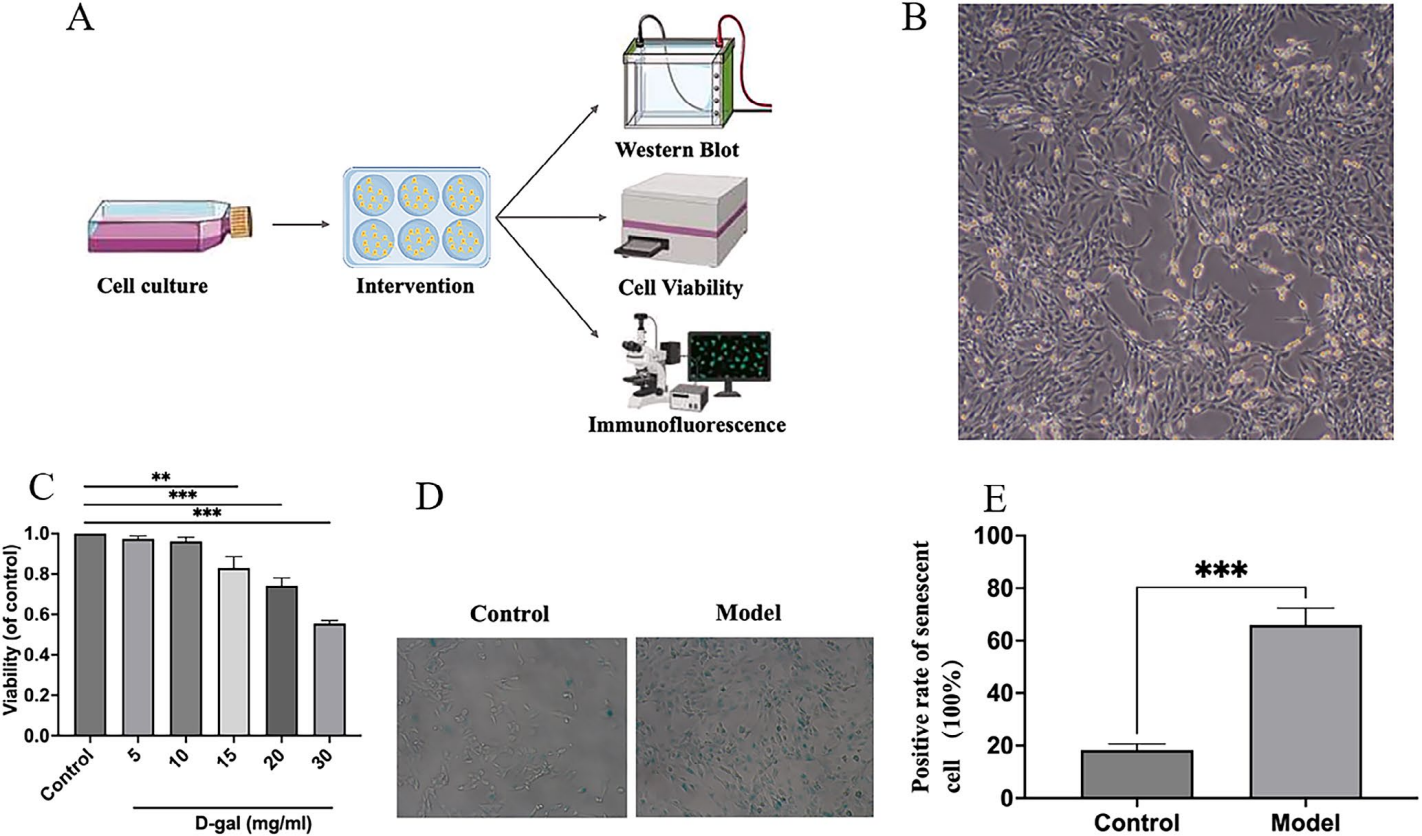
Flow chart and evaluation of cell model. (A) A simple flow chart of cell experiments. (B) Morphology of human brain microvascular endothelial cells (HBMECs) as observed under a light microscope. (C) D‐gal‐induced HBMECs viability was detected using the CCK‐8 assay. **p < 0.010, and ***p < 0.001 compared to control group, by one‐way ANOVA. (D) β‐galactosidase staining for control group and model group (15 mg/mL D‐gal). (E) β‐galactosidase staining positive rate count. ***p < 0.001 compared to control group, by two‐tailed *t*‐test. Columns represent five independent replicates. Error bars represent SD. CCK‐8, cell counting kit‐8; D‐gal, D‐galactose.

A senescence model in HBMECs was developed using D‐gal induction for 24 h, and the optimal concentration was ascertained through cell viability assays. The senescence model was validated by senescence‐associated β‐galactosidase (SA‐β‐Gal) staining. The experimental groups established for this study included the control group, the model group, and the drug‐containing serum intervention groups, which were further divided into low‐dose (BZBSL), medium‐dose (BZBSM), and high‐dose (BZBSH) subgroups of BZBS. Additionally, a pathway inhibitor group was included, using PI3K inhibitor LY294002 at a concentration of 5 μm/L based on previous studies and our preliminary experiments [22, 23]. To account for potential intergroup variability, the Control and Model groups received an equivalent volume of blank serum. The detailed cell experimental procedure is illustrated in Figure 1A. Raw data for all experiments are provided in the Supporting Information.

### 2.5 Optimal Intervention Concentration of D‐Galactose and Drug‐Containing Serum

After a 24‐h incubation period, varying concentrations of D‐galactose (5, 10, 15 mg/mL, 20 μmol/L, 30 mg/mL) and drug‐containing serum (5%, 10%, 15%, 20%) were introduced. Following an additional 24‐h incubation, 10 μL of Cell Counting Kit‐8 (CCK‐8) reagent was added to each well, and the plates were incubated in the dark for 2 h. Absorbance was subsequently measured at 450 nm using a microplate reader. Cell viability was calculated using the formula: Cell viability (%) = [(Absorbance of intervention group − Absorbance of blank group)/(Absorbance of control group − Absorbance of blank group)] × 100%.

### 2.6 SA‐β‐Gal Staining

Cells samples on plates were subjected to two washes with phosphate‐buffered saline (PBS). Subsequently, a fixative solution for β‐galactosidase staining was added and incubated at room temperature for 15 min. Following the removal of the fixative, the cells underwent three additional washes with PBS, each lasting 5 min. After the three washes with PBS, cells were incubated overnight using the staining solution at 37°C. Positive SA‐β‐gal staining cells were counted and the positive rate was calculated to evaluate the level of cell senescence.

### 2.7 Detection of Total Intracellular Nitric Oxide

The levels of NO in cells from each group were measured according to the instructions of the assay kit.

### 2.8 Western Blot Analysis

The cell samples were put into an ice‐cold radio immunoprecipitation assay (RIPA) buffer solution for homogenate preparation. After centrifugation, we used a BCA kit to measure the protein concentration of the lysates. After denaturation, protein samples were separated using electrophoresis on SDS‐PAGE gels and then transferred onto polyvinylidene fluoride (PVDF) membranes. Protein bands were blocked in QuickBlock Blocking Buffer for Western blot (Bio‐rad) under gentle rocking for 15 min at room temperature (RT) and incubated with primary antibodies (PI3K, 1:1000; Akt, 1:500; p‐Akt, 1:500; eNOS, 1:1000; GAPDH, 1:10,000) overnight at 4°C. Then, membranes were washed three times using TBST and probed with a 1:10,000 dilution of HRP‐conjugated secondary antibody for 1 h at RT. Immunoblots were visualized using ECL reagents. GAPDH was used as a normalization control. Each experiment was repeated at least five times. Band intensities were quantified using ImageJ software.

### 2.9 Immunofluorescence Detection

The cells were fixed using 4% paraformaldehyde for 10 min. Permeabilization was achieved with a strong permeabilization buffer for 10 min. Blocking of the cells was conducted with 10% goat serum for 1 h at 37°C. The cells were incubated with a primary antibody against eNOS (dilution 1:200) at 4°C overnight. Post‐incubation, the cells were treated with a goat anti‐rabbit fluorescent secondary antibody at room temperature for 1 h in the dark. DAPI was applied for nuclear staining. Finally, images were captured with a fluorescence microscope (Keyence) and were analyzed with ImageJ.

### 2.10 Statistical Analysis

GraphPad Prism (version 8.1.0) and IBM SPSS Statistics software (version 25) were used to plot and analyze data with suitable statistical tests. Continuous data are presented as the mean ± standard deviation (SD). For comparisons between two groups, an independent samples *t*‐test was utilized for normally distributed data, while the rank‐sum test was employed for non‐normally distributed data. Comparisons among multiple groups were assessed by one‐way ANOVA, followed by Bonferroni's multiple‐comparison test. A *p*‐value below 0.050 was deemed statistically significant.

## 3 Results

### 3.1 Establishment of an Aging Cell Model in HBMECs Induced by D‐Galactose

HBMECs were cultured in the endothelial cell proprietary medium supplemented with endothelial cell growth factor. Microscopic examination revealed that HBMECs adopted an elongated spindle morphology and organized into a cobblestone‐like pattern with localized vortex‐like formations (Figure 1B).

The results of the CCK‐8 assay demonstrated that a 24‐h exposure to varying concentrations of D‐galactose (5, 10, 15, 20, and 30 mg/mL) led to a progressive decrease in absorbance, indicating a concentration‐dependent reduction in cell viability. Compared to the control group, D‐galactose at a concentration of 15 mg/mL significantly reduced the viability of HBMEC (Figure 1C). Additionally, the positive rate of SA‐β‐gal staining cells was markedly higher in the 15 mg/mL D‐galactose treatment group relative to the control group (Figure 1D,E). Consequently, a concentration of 15 mg/mL D‐galactose was chosen for subsequent experimental procedures.

### 3.2 Optimal Concentration of Drug‐Containing Serum of BZBS

**FIGURE 2:**
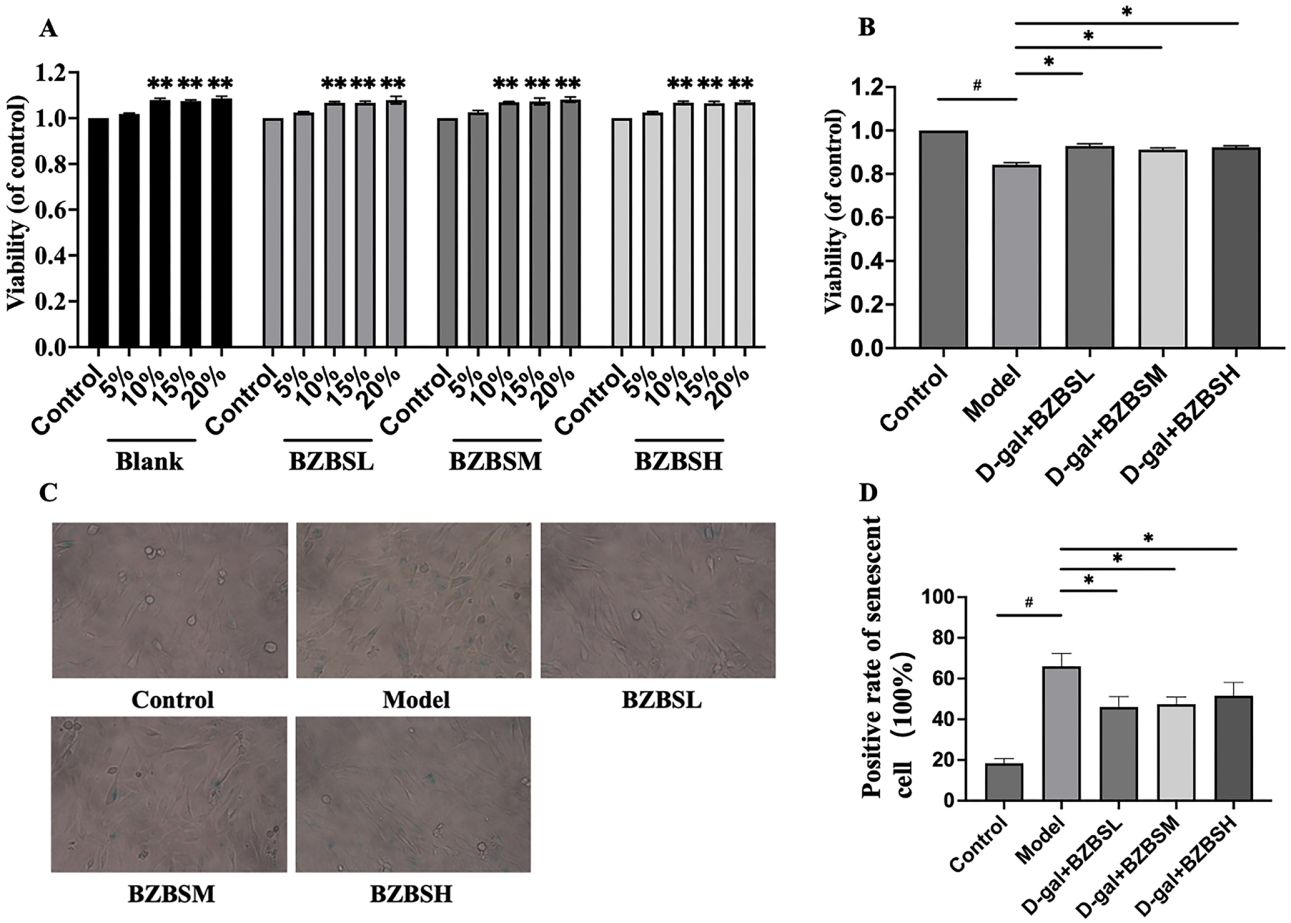
Effect of BZBS‐containing serum on senescent HBMECs. (A) Changes in cell viability after intervention with different concentrations of BZBS‐containing serum. **p < 0.010, and ***p < 0.001 compared to the control group, by one‐way ANOVA. (B) Changes in cell viability after intervention with D‐gal (15 mL/mL) and BZBS‐containing serum. ^#^ *p* < 0.050, *p < 0.050 compared to the model group, by one‐way ANOVA. (C) β‐galactosidase staining for different groups. (D) β‐galactosidase staining positive rate count. ^#^ *p* < 0.050, *p < 0.050 compared to the model group, by one‐way ANOVA. Columns represent five independent replicates. Error bars represent SD. BZBS, Bazi Bushen capsules; D‐gal, D‐galactose.

Following 24 h of treatment with varying concentrations of drug‐containing serum (5%, 10%, 15%, and 20%), cell viability was observed to increase when compared to the control group (high glucose basic medium without serum). However, no statistically significant differences in cell viability were detected among the different serum concentrations when the concentration exceeded 10% (Figure 2A). Given the potential for excessive differentiation at higher serum concentrations and adhering to principles of homogeneity and low‐dose safety, a 10% concentration of drug‐containing serum was selected for subsequent experiments.

### 3.3 Effects of BZBS on Aging HBMECs

Co‐culturing with D‐galactose and drug‐containing serum of BZBS for 24 h resulted in a significant increase in cell viability across all three drug‐containing serum groups (Figure 2B), as compared to the model group. Furthermore, the results of SA‐β‐gal staining (Figure 2C,D) demonstrated a significant reduction in the positive rate of senescent cells in the three drug‐containing serum groups relative to the model group.

### 3.4 Effects of BZBS on PI3K/Akt/eNOS Signaling Pathway

**FIGURE 3:**
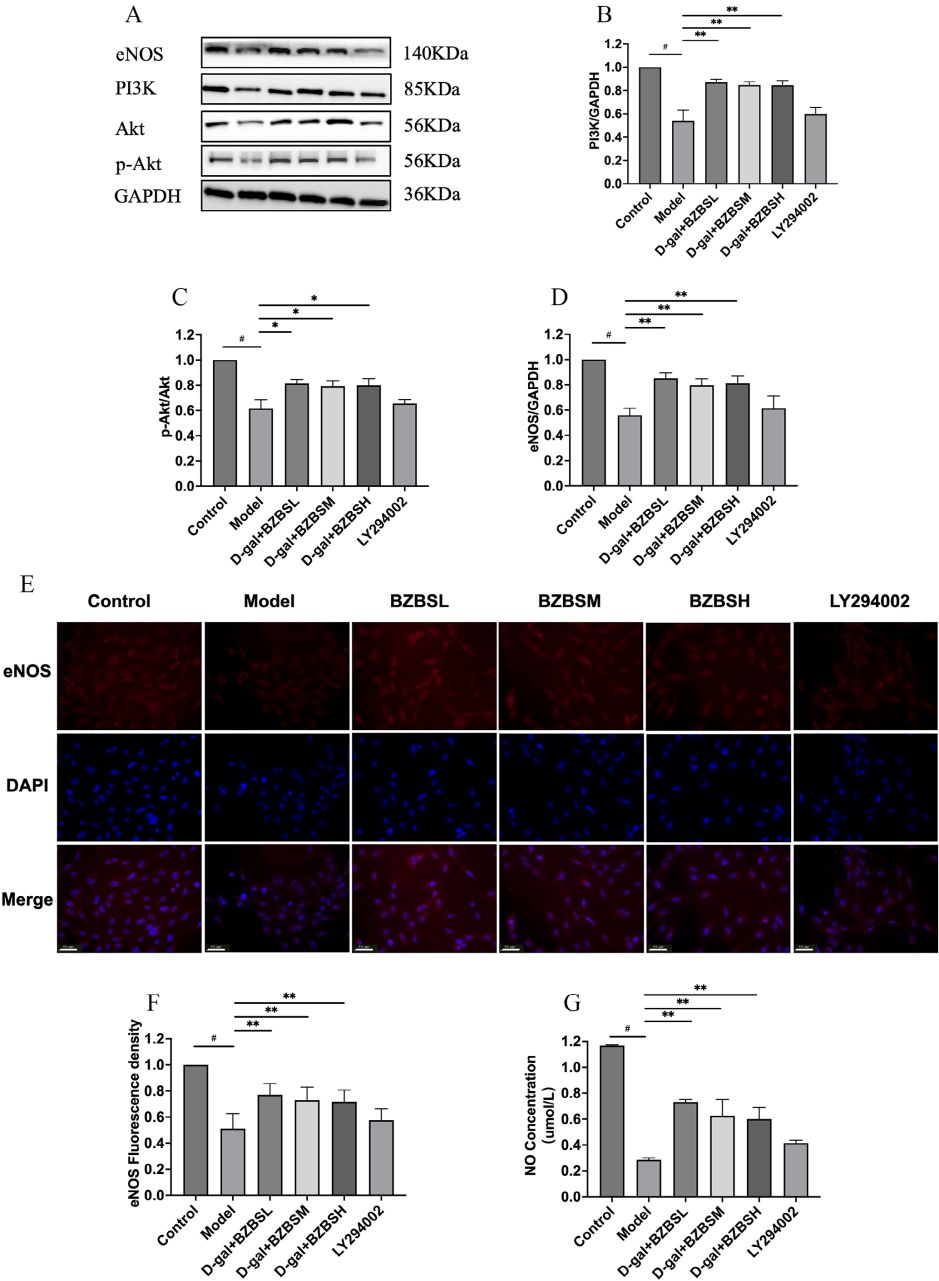
BZBS‐containing serum ameliorates the senescence of HBMECs through the PI3K/Akt/eNOS signaling pathway. (A) The blots of PI3K, Akt, p‐Akt, eNOS, and GAPDH. Changes in cell viability after intervention with D‐gal (15 mL/mL) for 24 h and BZBS‐containing serum. (B) The PI3K protein levels. (C) The ratio of p‐Akt/Akt. (D) The eNOS protein levels. (E) Fluorescence microscopy observation of eNOS. (F) The level of eNOS. (G) The concentration of NO. ^#^ *p* < 0.05, *p < 0.050, **p < 0.010 compared to the model group, by one‐way ANOVA. Columns represent five independent replicates. Error bars represent SD. Scale bars, 50 μm.

The expression levels of PI3K, Akt, p‐Akt, and eNOS proteins were assessed. Western Blot results (Figure 3A–D) showed that the model group exhibited a significant decrease in the expression levels of PI3K, Akt, p‐Akt, and eNOS compared to the control group. Notably, intervention with BZBS resulted in a significant increase in the expression levels of these proteins relative to the model group. To determine if the improvement in aging HBMECs is mediated through the PI3K/Akt/eNOS signaling pathway, we introduced the pathway inhibitor LY294002 (5 μM/L) into the BZBSL group. This approach was based on preliminary experiments and previous animal studies conducted by our research team. The addition of LY294002 attenuated the BZBS‐induced upregulation of PI3K, Akt, p‐Akt, and eNOS protein expression, resulting in no statistically significant difference compared to the model group.

Immunofluorescence staining results (Figure 3E,F) indicated a reduction in eNOS expression in the model group compared to the control group. The expression of eNOS was significantly elevated in all three drug‐containing serum groups relative to the control group. In contrast, there was no statistically significant difference in eNOS expression between the pathway inhibitor group and the model group.

The findings indicate that BZBS can upregulate eNOS protein expression in senescent HBMECs, potentially through the activation of the PI3K/Akt signaling pathway.

### 3.5 Effects of BZBS on NO Levels in Aging HBMECs

To further elucidate the mechanism by which the PI3K/Akt/eNOS signaling pathway enhances the function of aging HBMECs, NO content in the cell supernatants of all experimental groups was quantified (Figure 3G). The D‐galactose‐induced aging model group demonstrated a significant reduction in NO content compared to the control group. Conversely, the groups treated with drug‐containing serum of BZBS showed a statistically significant increase in NO levels relative to the model group. No significant difference in NO content was observed between the pathway inhibitor group and the model group.

## 4 Discussion

This study demonstrated that drug‐containing serum of BZBS reduces the rate of SA‐β‐Gal positive cells in HBMECs, suggesting that BZBS may delay the aging process of HBMECs. Experimental results indicated that the model group exhibited decreased levels of PI3K, Akt, p‐Akt, and eNOS, along with reduced NO content. Intervention with BZBS increased the expression of PI3K, Akt, p‐Akt, and eNOS, as well as elevated NO content. A comprehensive analysis indicates that the low‐dose drug‐containing serum group exhibited the most significant improvement. In our study, we found that the group of BZBSL achieves the best therapeutic effect among the three doses. Pharmacologically, saturation of metabolism may partly explain a nonlinear dose–response relationship. The application of the PI3K inhibitor LY294002 abrogated the beneficial effects of BZBS on PI3K, p‐Akt, Akt, eNOS, and NO, suggesting that the PI3K/Akt/eNOS signaling pathway is a key mechanism through which BZBS enhances the function of aging HBMECs.

The PI3K/Akt/eNOS signaling pathway is essential for endothelial cell function, orchestrating various biological processes including cell growth, metabolism, and apoptosis [24]. PI3K is a pivotal molecule in growth factor signaling pathways, with Akt serving as a crucial downstream target that mediates cell growth, proliferation, and apoptosis. The activation of PI3K facilitates the phosphorylation of Akt, which subsequently modulates the activation and expression of various downstream effectors. NO is predominantly synthesized by eNOS, an enzyme extensively expressed in endothelial cells. The activation of eNOS augments the production and release of NO, thereby contributing to vascular protection. As a primary substrate of Akt, eNOS can be activated by Akt to promote NO release, which subsequently promotes vasodilation, reduces cellular apoptosis, and inhibits the adhesion of leukocytes and platelets to the vascular endothelium [6]. Activation of the PI3K/Akt/eNOS signaling pathway has been demonstrated to enhance eNOS activity and NO release, thereby ameliorating vascular dysfunction [25].

TCM presents a comprehensive framework of theories and therapeutic modalities addressing the aging process. Aging is fundamentally linked to “kidney deficiency” in TCM theories, characterized by the progressive depletion of kidney essence and an imbalance of yin and yang, which subsequently leads to the gradual deterioration of organ function. Age‐related kidney deficiency is intrinsically connected to the natural aging process. This theory provides a basis for the development of kidney‐tonifying interventions aimed at mitigating the effects of aging. Clinical research has demonstrated that kidney‐tonifying interventions can ameliorate clinical symptoms and signs in patients with cerebrovascular diseases, potentially by enhancing endothelial cell function, promoting neovascularization, and facilitating the establishment of collateral circulation.

BZBS is traditionally used as a kidney‐tonifying and anti‐aging drug. Research has demonstrated that BZBS can augment the resilience of *Caenorhabditis elegans* to external stimuli, extend its lifespan, enhance the frailty index and overall phenotype in aging mice, and delay aging by maintaining telomerase activity, upregulating SIRT6 protein, and downregulating the aging‐associated protein p53 [17, 18, 21]. Fingerprint analysis of BZBS revealed that it contained a variety of PEs [17]. Estrogen exerts potent vasculoprotective effects that are partly mediated by inhibiting inflammation and apoptosis [26]. The PEs composition of BZBS may underlie the beneficial effects of BZBS to protect against aging and is worthy of further exploration. Additionally, the active ingredients of ginseng, including saponins, polysaccharides, and active peptides, have antioxidant, anti‐apoptotic, neuroprotective, and aging‐delaying effects [27, 28, 29, 30, 31].

Although the study provides important insights, several limitations must be acknowledged. First, BZBS is a complex molecular mixture, and its complete composition remains partially unidentified; thus, the precise components of BZBS still require further investigation. Second, this study was conducted in vitro, and the experimental methods to elucidate the mechanism are relatively limited. Future research should incorporate multi‐omics and high‐throughput sequencing technologies to reveal the mechanism of BZBS in regulating aging and preventing age‐related diseases from multiple perspectives [32]. Third, it is imperative to conduct clinical trials to assess the safety and efficacy of BZBS in human populations for age‐associated vascular diseases.

In conclusion, this study indicates that BZBS could delay the aging of endothelial cells, which may be related to the activation of the PI3K/Akt/eNOS signaling pathway. These findings provide novel insights and identify potential targets for clinical interventions through the application of TCM.

## Author Contributions

Jinzhou Tian and Jing Shi designed and supervised the study. Lulu Yao conducted the experiments and collected the data. Lulu Yao, Fuyao Li, Jingnian Ni, Mingqing Wei, and Ting Li analyzed the data. All authors participated in the manuscript writing and revision.

## Ethics Statement

All experimental procedures were approved by the Animal Ethics Review Board of Beijing University of Chinese Medicine (Ethics Review No. 2023‐041307‐2102).

## Conflicts of Interest

The authors declare no conflicts of interest.

## Supporting information


Appendix S1

## Data Availability

The datasets and figures supporting the conclusions of this article are included within the article and its additional files.
