# Supplementary material for: Effect of Bazi Bushen Capsule on D‐Galactose‐Induced Human Endothelial Cell Senescence Through PI3K/Akt/eNOS Signaling Pathway
Source: Aging Med (Milton). 2025 Jun 11;8(3):258–66. doi: 10.1002/agm2.70031 (PMC12226422; doi:10.1002/agm2.70031)
Supplement: Supplementary file 1 — Appendix S1 [file AGM2-8--s001.docx]

*Supplementary material*

Table 1. The components of BaZiBuShen capsule (BZBS).

| Chinese name | Latin name | Use part |
| --- | --- | --- |
| Tusizi | Cuscutae | Semen |
| Gouqizi | Fructus Lycii | Fructus |
| Yinyanghuo | Epimedii Folium | Stem and leaf |
| Nanwuweizi | Fructus Schisandrae Sphenantherae | Fructus |
| Shechuangzi | Fructus Cnidii | Fructus |
| Jinyingzi | Fructus Rosae Laevigatae | Fructus |
| Fupenzi | Fructus Rubi | Fructus |
| Jiucaizi | Semen Allii Tuberosi | Semen |
| Chuanlianzi | Fuctus Toosendan | Fuctus |
| Bajitian | Morindae Officinalis | Radix |
| Roucongrong | Herba Cistanches | Stem |
| Shengdihuang | Rehmanniae Recens | Radix |
| Chuanniuxi | Radix Cyathulae | Radix |
| Renshen | Ginseng | Radix |
| Lurong | Cervi Cornu Pantotrichum | Baby horn |
| Haima | Hippocampus | Dried body |

Table 2. Key resources.

| **Reagent and resource** | **Species** | **Dilution** | **Manufacture** | **Cat. Number** |
| --- | --- | --- | --- | --- |
| **Antibody** | | | | |
| PI3K | Rabbit | 1:1000 | Abcam | Ab227204 |
| Akt | Rabbit | 1:500 | Abcam | Ab18785 |
| p-Akt | Mouse | 1:500 | Abcam | Ab38449 |
| eNOS | Rabbit | 1:1000 | Abcam | Ab300071 |
| GAPDH | Rabbit | 1:10000 | Abcam | ab181602 |
| Alexa Fluor 488 | Goat | 1:1000 | Bioss | bs0295G |
| Alexa Fluor 594 | Goat | 1:1000 | Bioss | bs0296G |
| **Reagents** | | | | |
| DMEM High Glucose Medium | - | - | Hyclone | AG29719232 |
| Endothelial Cell Medium | - | - | ScienCell | 1001 |
| Trypsin solution | - | - | Gibco | 25200072 |
| Fetal bovine serum | - | - | Gibco | 12483020 |
| Normal goat serum | - | - | Solarbio | SL038 |
| QuickBlock Blocking Buffer | - | - | Bio-rad | 12010020 |
| **Chemicals** | | | | |
| D-galactose | - | - |  | G0750 |
| LY294002 | - | - | MedChemExpress | HY-10108 |
| **Critical commercial assays** | | | | |
| Senescence-Associated β-Galactosidase Assay Kit | - | - | Beyotime | C0602 |
| BCA Protein Assay Kit | - | - | Beyotime | P0010 |
| Total Nitric Oxide Assay Kit | - | - | Beyotime | S0024 |
| Immunostaining Permeabilization Solution | - | - | Beyotime | P0095 |
| CCK-8 Assay Kit | - | - | Analysis Quiz | AQ-308 |
| SuperSignal™ ECL Chemiluminescent Substrate Kit | - | - | Proteintech | PK10003 |
| Protein Pre-stained Marker | - | - | Proteintech | PL00002 |
| The Omni-Easy One-Step PAGE Gel Preparation Kit | - | - | Yamei | PG212 |
| **Experimental models** | | | | |
| Human brain microvascular endothelial cells | - | - | Shanghai Zhongqiao Xinzhou | ZQ0961 |
| SD rats | - | - | Vital River | 101 |
